# Sedentary Behavior in Older Patients before and after Total Hip Arthroplasty: A Prospective Cohort Study

**DOI:** 10.3390/healthcare8030346

**Published:** 2020-09-18

**Authors:** Burkhard Moellenbeck, Frank Horst, Georg Gosheger, Christoph Theil, Leonie Seeber, Tobias Kalisch

**Affiliations:** 1 Department of Orthopedics and Tumor Orthopedics, Muenster University Hospital, 48149 Muenster, Germany; burkhard.moellenbeck@ukmuenster.de (B.M.); georg.gosheger@ukmuenster.de (G.G.); christoph.theil@ukmuenster.de (C.T.); leonie-seeber@hotmail.de (L.S.); 2 Department of Orthopedics and Traumatology, St. Josef-Stift Sendenhorst, 48324 Sendenhorst, Germany; horst@st-josef-stift.de

**Keywords:** hip osteoarthritis, arthroplasty, physical activity, sedentary behavior

## Abstract

*Objective:* To compare the habitual sedentary behavior (SB) and physical activity (PA) of older hip osteoarthritis patients before and after elective arthroplasty. *Methods:* SB, PA and joint-specific disability of 16 patients (68.9 ± 6.8 years) were assessed by accelerometry and questionnaires before and 9 months after arthroplasty. *Results:* All patients reported substantial postoperative improvements of their joint-related complaints (*p* ≤ 0.001). Accelerometry showed changes in neither daily SB (10–60 min sedentary bouts, *p* ≥ 0.569) nor in PA (steps, time in mild-to-vigorous activity and energy expenditure, *p* ≥ 0.255). Correlation analyses revealed that patients with severe preoperative disability showed a decrease in sedentary time, which was the opposite in patients with mild preoperative disability. *Conclusion:* SB and PA do not necessarily change after arthroplasty in older orthopedic patients. Even longer bouts of uninterrupted sitting, which are detrimental to health, do not decrease. Preoperative patient education is recommended to foster behavioral changes following elective arthroplasty.

## 1. Introduction

Osteoarthritis (OA) is an age-related and progressive disease, causing pain, loss of function and disability in those affected [1]. OA is most prominent in lower-extremity joints like the knee and the hip [2]. Due to the increasing age of western populations and the wider adoption of a physically inactive lifestyle, a further increase in the incidence of OA can be expected [3]. Following conservative treatment, total hip arthroplasty (THA) is indicated in end-stage OA [4]. THA followed by rehabilitation usually leads to complete freedom from pain, a significant improvement of patient health related quality of life, the restoration of joint function and significant improvements in physical functioning [5,6,7]. In addition to these effects of surgery, most OA patients also expect an improvement of their physical activity (PA) [8,9]. Using objective methods like body-worn accelerometers, it could be demonstrated that an improvement of PA does not necessarily occur, not even within a period of 6–12 months postarthroplasty [10,11]. Therefore, serious deficits in lower limp functional performance and walking safety may occur in OA patients in the long run [12].

PA has a positive effect in avoiding comorbidities that are modifiable by activity, as well as improving physical performance, the level of pain and depression [13], which should be a goal in treating OA patients, as they are frequently not provided with common PA recommendations [14]. However, reduced PA is not the only reason for the development of negative health-conditions in older patients. The effects of an inactive lifestyle, characterized by sedentary behavior (SB, i.e., low energy expenditure occurring in a sitting, reclining or lying position), are supposed to be at least as risky [15]. Today, up to 60% of an adult’s waking hours are sedentary, which, in extreme cases, can be as much as 10 h/day [16]. Excessive SB is associated with an increase in cardiovascular disease and overall mortality. This is apparently even true for individuals who meet the common recommendations for PA [17]. Therefore, SB represents a separate and clinically important aspect of a patient’s activity profile and should no longer be considered only as the extreme lower end of PA [16]. Particularly periods of prolonged and uninterrupted sitting (‘bouts’) have harmful effects on the cardiovascular system, suggesting that not only the total time spent sedentary is a health risk, but even more the way it is composed. Data from epidemiological studies confirmed these findings by reporting that people who often sit continuously (e.g., due to occupational activities) have a worse cardiometabolic profile than those who frequently interrupt their sitting time [18].

Older adults are the most sedentary age group [19], and are also commonly affected by OA, which puts this group at particularly high risk [20,21]. Based on the finding that interrupting sedentary activities has a positive effect on the health of older adults and improves physical function [22], we here aim to precisely assess the time spent in sedentary bouts of different length, as well as the time between those bouts (i.e., ‘sedentary breaks’). So far, only little is known about the effects of arthroplasty on the SB of older patients suffering from hip OA. Our hypothesis states that even if the habitual PA of the OA patients might not change following arthroplasty, there should be a change in their SB. Specifically, we expect a reduction in the uninterrupted sedentary bouts. To test this hypothesis, four different time periods of uninterrupted sitting (i.e., 10-, 20-, 30- and 60 min) were assessed by accelerometry before and after arthroplasty in a group of older OA patients and analyzed for significant changes.

## 2. Materials and Methods

Data were drawn from a larger, longitudinal study investigating the behavioral alignment of PA and SB in hip and knee OA patients and their spouses. Comprehensive information on the recruitment procedure and eligibility criteria have therefore been published previously [23]. The study was approved by the local Ethics Committee on and registered in a clinical trials register (German Clinical Trials Register: DRKS00014292) (2017-613-f-S).

### 2.1. Participants

A range of information regarding participant characteristics and demographics was collected as part of the parent study. This included age, education, body mass index and comorbidities (Table 1). Participants were recruited in the two orthopedic departments involved in the study (located in NRW, Germany). The criteria for inclusion were end-stage hip OA, age between 50 and 85 years and sufficient language skills in German or English to understand the objectives and requirements of the study. Exclusion criteria were defined as any nonorthopedic condition (e.g., rheumatic, musculoskeletal, cardiovascular, neurologic, etc.) significantly limiting PA or causing increased SB in everyday life [23]. In detail, data of 16 patients suffering from OA for approx. 5.8 years (median; interquartile range IQR: 0.9–7.2 years) (first medical treatment of OA-related complaints) were analyzed before and 8.9 ± 2.3 months after elective THA. All patients took part in inpatient rehabilitation (2–3 weeks) following surgery.

### 2.2. Procedures

Physical activity was assessed by tri-axial ActiGraph wGTX3-BT (Firmware 1.9.2) activity monitors (ActiGraph LLC, Fort Walton Beach, FL, USA) that were worn on the waist and close to the body’s center of mass using elastic belts. The patients were instructed to remove the monitors only for water-based activities such as bathing and swimming. The monitors were initialized as per the manufacturer’s instructions. Data were downloaded using the ActiLife Software provided by the manufacturer (ver. 6.13.4, ActiGraph LLC, Fort Walton Beach, FL, USA). The minimum wear time was set to 10 h per day for at least 4 days; this is required to obtain reliable PA and SB estimates [24]. The monitor sampling frequency was set at 100 Hz and the epoch length at 10 s. Nonwear times were automatically detected and excluded from analyses [25]. Based on previous knowledge about this cohort, night hours (11:01 p.m.–05:59 a.m.) were excluded from the data acquisition by default.

The analysis of the patients’ PA was based on the following parameters: Average daily number of steps (‘Steps’), time in moderate-to-vigorous PA (‘MVPA’) and the PA energy expenditure (‘PAEE’ in kcal) using the “Freedson vector magnitude equation” (VM3) which utilizes data from all three axes of motion [26]. The analysis of the patients’ SB was based on the average daily time spent in ‘sedentary bouts’ (i.e., 10-, 20-, 30- or 60 min with an activity in the strict range of 0–99 counts per minute, which corresponds to metabolic equivalents of task (METs) in the range of 1.0–1.5 [27]. Additionally, the time between those bouts was quantified as average daily ‘sedentary breaks’ (i.e., 10-, 20-, 30- or 60 min with an activity ≥100 counts per minute). Sedentary bouts and breaks were summed across each compliant day (≥10 h of wear) and then averaged across all of a participant’s compliant days to derive average per-day values. By way of explanation, it should be noted that in this calculation, it may happen that the average time per day spent in a particular bout is below the respective minimum for the corresponding bout.

All patients reported their age, sex, height and weight, which were used to calculate their daily basal metabolic rate (BMR, amount of calories expended at rest) according to the Harris-Benedict energy equation [28].

The Lequesne index (LI) of severity for osteoarthritis covers specific symptoms and physical functional disability in patients suffering from hip or knee osteoarthritis [29]. It aggregates symptoms and function, where ‘pain’ is analyzed by five items, ‘maximum distance walked’ by two items and ‘activities of daily living’ by four items. The score ranges from 0 to 24 (maximum pain/disability) and is scored as the sum of all questions where 0 is no handicap, 1–4 equals a mild handicap, 5–7 equals a moderate handicap, 8–10 equals a severe handicap, 11–13 equals a very severe handicap, and 14 equals an extremely severe handicap.

The Hip Osteoarthritis Outcome Score (HOOS) is an instrument to assess the patients’ opinions about their hip and associated problems in the context of joint injury or osteoarthritis during the last week [30]. It consists of five subscales: ‘pain’, ‘other symptoms’, ‘everyday functionality’, ‘function in sport and recreation’ and ‘knee related quality of life’. Standardized answer options are given, and each question is assigned a score from 0 to 4. A normalized score (100 indicating no symptoms and 0 indicating extreme symptoms) is calculated for each subscale. A total score was not validated and is not recommended [31]. 

### 2.3. Statistical Analyses

After data collection, all patient data were pseudonymized. All analyses were performed with SPSS (Version 26, SPSS Inc., Chicago, IL, USA), with the significance level set at α = 0.05. Parametric and nonparametric statistical methods were used depending on the data distribution (testing for normality was done using the Shapiro-Wilk Test). Data were reported as mean and standard deviation (SD)/95% confidence interval (CI) or median and interquartile range (IQR). Differences between pre- and post-arthroplasty data were investigated using paired t-tests (or Wilcoxon signed-rank tests). Coherence between survey and accelerometric data were investigated using Spearman’s rank correlation analyses.

## 3. Results

### 3.1. Sample Characteristics

The characteristics of the 16 participants are detailed in Table 1. With 9 male and 7 female patients, the gender ratio in the group was almost balanced, and the subjects were aged between 55 and 81 years.

### 3.2. Patient Reported Joint Limitations

**Figure 1:**
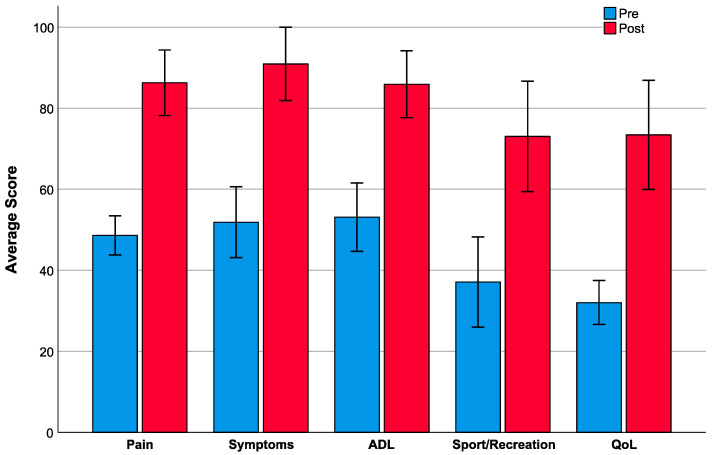
Comparison of HOOS subscale scores before (pre, blue bars) and after arthroplasty (post, red bars) in all patients. All outcomes were reported as means, error bars represent 95% CI. A score of 100 represents the best possible outcome score. Paired *t*-tests revealed significant differences between pre and post condition for all subscale scores (*p* ≤ 0.001). ADL = activities of daily living; QoL = quality of life.

All patients reported substantial postoperative improvements of their joint-related complaints in the HOOS questionnaire. In detail, the patients reported an average increase of 37.7 ± 18.4 points (*p* ≤ 0.001) for the subscale score Pain, 39.1 ± 19.2 points (*p* ≤ 0.001) for the subscale score Symptoms, 32.8 ± 22.3 points (*p* ≤ 0.001) for the subscale score ADL, 35.9 ± 35.1 points (*p* ≤ 0.001) for the subscale score Sport/Recreation and 41.4 ± 26.1 points (*p* ≤ 0.001) for the subscale score QoL. In this context, the criteria of ‘clinically meaningful changes’ (MDC_90_; i.e., minimal detectable change based on a confidence level of 90%) of the subscale scores were achieved by the majority of patients (Pain, Symptoms and ADL: 15/16 patients (MDC_90_ = 6.09; 6.55; 6.55); Sport/Recreation: 13/16 patients (MDC_90_ = 8.63); QoL: 14/16 patients (MDC_90_ = 8.22) [32] (Figure 1).

### 3.3. Physical Activity

**Figure 2:**
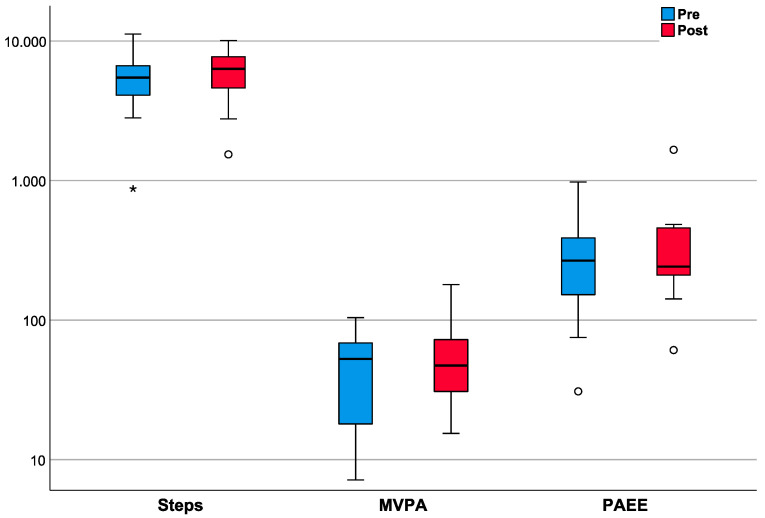
Comparison of the patients’ pre- (blue bars) and post- arthroplasty PA (red bars) as assessed by the daily number of steps (Steps), the time in moderate-to-vigorous PA (MVPA) and the PA related energy expenditure (PAEE). The plots represent the median (thick horizontal lines), the values in the IQR (inside the boxes) and the remaining 50% of the data (whiskers). Outliers and extreme outliers (i.e., 1.5 × IQR) are represented by circles and stars. For none of the three examined parameters a significant change could be determined (*p* ≥ 0.255). For a better overview of the 3 examined parameters the x-axis was scaled logarithmically.

All patients met the defined minimum usage of accelerometry both before (6.3 ± 1.0 days) and after arthroplasty (5.4 ± 1.2 days). PA was quantified by the daily number of steps, the time in MVPA and the PAEE, which showed a high degree of positive intercorrelation (r ≥ 0.621; *p* ≤ 0.010) before THA. The daily number of steps changed from 5465.21 (median; IQR: 3972.55–6752.65) before arthroplasty to 6311.05 (median; IQR: 4460.87–7761.26) postarthroplasty (*p* = 0.278). There was a significant positive correlation between the pre- and post- operative number of steps (r = 0.665; *p* = 0.005). The daily time in MVPA changed from 52.93 min (median; IQR: 16.12–69.24) to 47.36 min (median; IQR: 29.25–79.91) (*p* = 0.278) with a significant positive correlation between the pre- and post- operative daily activity (r = 0.709; *p* = 0.002). Patient PAEE changed from 267.16 kcal (median; IQR: 141.15–393.68) to 242.30 kcal (median; IQR: 208.94–458.08) (*p* = 0.255), showing a strong trend towards a positive correlation between pre- and post- arthroplasty energy expenditure (r = 0.497; *p* = 0.050). In summary, it can be stated that according to accelerometry 8.91 ± 2.30 months after THA, no changes in PA were observed in the OA patients (Figure 2).

### 3.4. Sedentary Behavior—Time in Bouts

**Figure 3:**
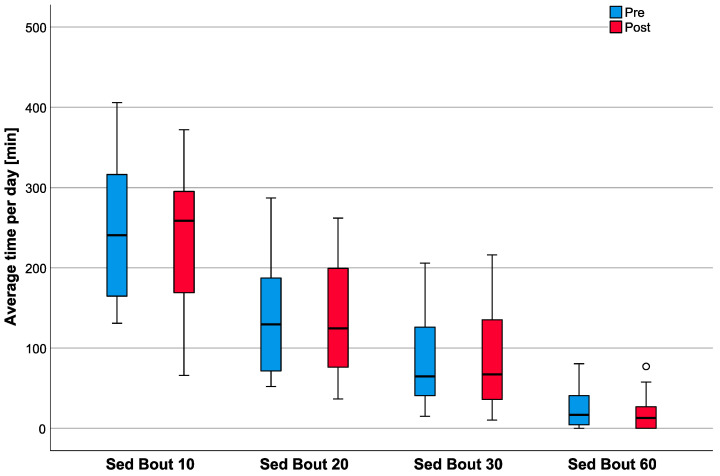
Comparative illustration of patient pre- (blue bars) and post- arthroplasty SB (red bars), as assessed by the average daily time spent in sedentary bouts of 10-, 20-, 30- and 60-min length. The plots represent the median (thick horizontal line), the values in the IQR (inside the box) and the remaining 50% of the data (whiskers). Outliers (i.e., 1.5 × IQR) are represented by circles. There were no significant changes from the pre- to the post- arthroplasty measurement for any bout duration (*p* ≥ 0.569). Correlation analyses revealed a significant coherence between the patients’ pre- and post- arthroplasty time in 10-min bouts (r = 0.553; *p* = 0.026) but not for 20, 30- and 60-min bouts (r ≤ 0.374; *p* ≥ 0.154).

The detailed investigation of patient SB was based on the average time spent in four specific sedentary bouts (10-, 20-, 30- and 60-min length) per day. These times showed a high degree of intercorrelation (r ≥ 0.688; *p* ≤ 0.017) before THA. In detail, the time spent in 10-min bouts changed from 240.59 min (median; IQR: 159.88–317.92) to 258.59 min (median; IQR: 168.43–295.44) per day (*p* = 0.796) with a positive correlation between pre- and post- arthroplasty data (r = 0.553; *p* = 0.026). The time spent in 20-min bouts changed from 129.48 min (median; IQR: 70.93–187.46) to 124.40 min (median; IQR: 74.66–203.98) per day (*p* = 0.679). The time spent in 30-min bouts changed from 64.58 min (median; IQR: 36.52–126.69) to 67.19 min (median; IQR: 33.59–139.43) per day (*p* = 1.000). Finally, the time spent in 60-min bouts changed from 16.81 min (median; IQR: 2.17–41.34) to 12.98 min (median; IQR: 0.0–27.37) per day (*p* = 0.569) (Figure 3). For the 20-, 30- and 60-min bouts, no correlation between pre- and post- arthroplasty data could be found (r ≤ 0.374; *p* ≥ 0.154).

**Figure 4:**
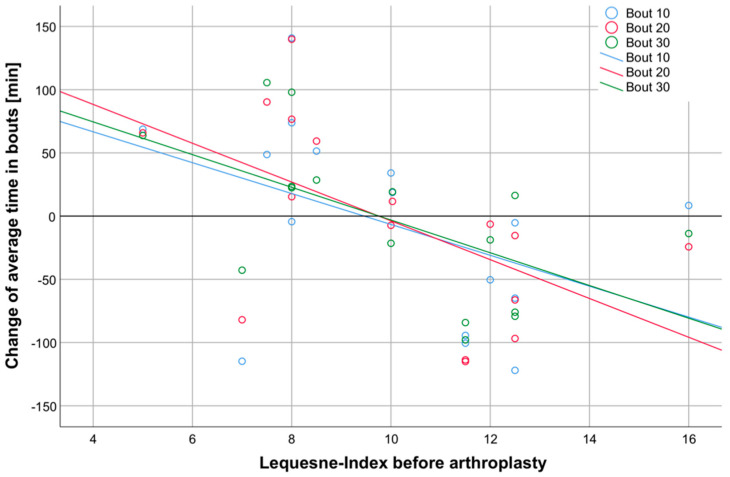
Correlation analyses of the changes in daily sedentary time and the preoperative LI score. For the 10-, 20-, and 30-min sedentary bouts, there was an increase in time found for patients with a lower prearthroplasty LI (LI < 10) and a decrease in time found those with a higher prearthroplasty LI (LI > 10) (r ≥ −0.493, *p* ≤ 0.026). This coherence could not be revealed for data of the 60-min bouts (r = −0.330, *p* = 0.106, graph not shown).

A possible relationship between the preoperative OA-related disability as quantified by the LI and patients’ individual changes in SB was investigated subsequently. For the 10-min, 20-min and 30-min bouts, mostly patients with severe preoperative disability (LI > 10) showed a decrease in sedentary time, whereas patients with mild preoperative disability (LI < 10) mostly showed an increase in sedentary time (10-min bouts: r = −0.493; *p* = 0.026; 20-min bouts: r = −0.597; *p* = 0.007; 30-min bouts: r = −0.585; *p* = 0.009). For the 60-min bouts, however, no such correlation could be demonstrated (r = −0.330; *p* = 0.106) (Figure 4).

### 3.5. Sedentary Behavior—Breaks between the Bouts

The second parameter by which the SB of the patients was investigated pre- and post- arthroplasty was the daily active time between two specific bouts, i.e., the sedentary breaks. The patients’ time spent in 10-min-breaks changed from 1074.72 min (median; IQR: 986.57–1209.14) to 1047.43 min (median; IQR: 949.04–1136.90) (*p* = 0.278). The time in 20-min-breaks changed from 1113.79 min (median; IQR: 901.75–1239.86) to 1106.78 min (median; IQR: 975.82–1189.96) (*p* = 0.918). The time spent in 30-min-breaks changed from 1048.80 min (median; IQR: 705.73–1287.71) to 995.47 min (median; IQR: 724.68–1134.48) (*p* = 0.535). Finally, the time spent by patients in 60-min-bouts changed from 1.09 min (median; IQR: 0.0–873.43) to 0.0 min (median; IQR: 0.0–454.93) (*p* = 0.272). In accordance with the preceding sedentary bout analyses, no significant correlations between the patients’ individual times in specific sedentary breaks before and after the THA could be found (r ≤ 0.415; *p* ≥ 0.110).

Finally, the relationship between the preoperative OA-related disability and the patients’ individual changes in specific sedentary breaks was investigated. In patients with severe preoperative disability (LI > 10), the time spent in 10- and 20-min sedentary breaks increased, whereas patients with mild preoperative restrictions (LI < 10) displayed a decrease in sedentary break time (10-min breaks: r = 0.688; *p* = 0.002; 20-min breaks: r = 0.471; *p* = 0.033). This coherence was, however, not found for 30-min and 60-min breaks (r ≤ 0.379, *p* ≥ 0.074). 

## 4. Discussion

Although SB in hip OA patients has been examined before, previous studies have often only reported daily total times or tolerated temporal interruptions in sedentary bouts, which may have masked small changes in patient postarthroplasty behavior (see [33] for review). For this reason, the main objective of the current study was to investigate SB by means of different bout lengths and strict criteria.

Almost 9 months after surgery, the patients in the present study reported significant and meaningful improvements in their joint-related situation, not only for pain, joint symptoms and quality of life, but also for activities of daily living, recreational activities and sport. As a result, one might have expected that this subjective rating would be confirmed by accelerometry measurements; however, this was not the case, thus confirming previous findings [4]. The patients’ PA, which was assessed by the daily number of steps, the time in MVPA and the PAEE showed no changes after THA. For each of these three parameters, a significant correlation between the prearthroplasty and postarthroplasty performance could be demonstrated, indicating that the individual patients remained at their respective performance levels. These results were in line with previous findings that demonstrated no or only minor changes in PA of OA patients 6–12 months postarthroplasty [11,34].

The investigation of the patients’ SB according to different bout lengths matched the results of the PA described above, in that no changes following THA could be demonstrated. In contrast to the investigated PA parameters, patient individual postarthroplasty times in longer sedentary bouts (20-, 30- and 60 min) were not correlated with the prearthroplasty-situation. This might be a first indication that although the negative outcome of PA and SB is comparable in general, SB should be investigated separately and in more detail. Our findings support the statements of other studies, which do not regard SB as the lowest end of PA, but rather, as an independent construct [35,36]. In order to be able to detect even small changes in SB, we applied a strict bout criterion in our study. In contrast to previous studies that allowed a temporal interruption of bouts and used uniaxial sensor data, a bout was terminated immediately when the activity with a limit of 100 triaxial counts/minute was exceeded for the first time. For this reason, we describe a SB that differs from that described in other studies on OA patients, where it was shown that the patients spent most of their daily sedentary time in long bouts (≥30 min) [37]. The used bout criterion improves the detection of interruptions of sedentary activities, which would have been expected in OA patients after THA. This would have resulted in a shift of sedentary time from longer bouts to shorter ones, but this was also not the case. These findings were confirmed by complementary analyses of the sedentary breaks, which also did not change postarthroplasty. Whether a short-term interruption of a bout by getting up or walking around is already beneficial to health [38], or whether higher intensity PA (e.g., MVPA) is required to avoid the harmful influence of sedentary behavior, is still under discussion [18,39]. For older people, however, it is clear that reducing the number and length of sedentary bouts has a positive effect on health [22].

The question of why some studies were able to demonstrate a change in patient activity levels following arthroplasty while others were not has been repeatedly discussed [40]. In addition to the fact that postoperative rehabilitation programs vary across clinical settings and across countries, there is also a need for a more detailed investigations of SB, i.e., going beyond total sedentary time [37]. The data presented here give us reason to suspect that the extent of preoperative disability may influence the way in which patient SB changes postarthroplasty. Patients with minor preoperative disabilities showed a slight increase in SB, as well as a reduction in sedentary break times, whereas the situation was exactly the opposite in the other patients. This finding has yet to be confirmed in larger patient populations, but if, for whatever reason, patients with minor joint-related disabilities tend to sit more postoperatively, and those who were severely disabled do the opposite, group effects could be masked or at least strongly depend on the composition of the examined cohort.

This study has some limitations. First of all, only a small group, i.e., 16 patients, conveniently taken from a larger cohort [23], was included in the study. This small number might reduce the odds of detecting true effects. Nevertheless, the analyses are based on paired data from patients whose age, BMI and activity levels are comparable to other OA studies, and the negative results presented here are quite clear. Secondly, although all patients participated in a structured rehabilitation program, it is unfortunately not known how long the intervention lasted for the individual patients. Finally, some limitations should be recognized that can occur when examining everyday activities with accelerometers. For technical reasons, not all types of PA (e.g., cycling, climbing stairs) can be correctly recorded. In addition, patients may somehow adjust their usual behavior in response to the use of an accelerometer (e.g., due to social desirability), although this effect should generally be short-lived [41].

Why is the further investigation of SB in older OA patients so important? First, because it has been shown that an increase of sedentary activities in old age is associated with a loss of everyday skills, which cannot be compensated for by PA [42]. Second, because it was demonstrated that the sedentary lifestyle of OA patients is adopted by their partners [23], and thus, it could affect a much larger group of people than previously thought. The development of noncommunicable diseases and the possible loss of independence in everyday life due to excessive SB is a great challenge, not only for individuals, but also for the health care systems [43].

Considering that THA is one of the most frequently performed operations worldwide [44], and that these numbers will continue to rise in view of an ageing population [45], further research is needed to improve outcomes after surgery. For older patients, THA is a stressful event [46], and in light of their poor physical function prior to surgery and the likelihood of further decline during hospitalization, it is hypothesized that prehabilitation programs could improve outcomes after total joint replacement surgery [47,48].

## 5. Conclusions

Here, it was demonstrated that THA in a sample of German hip OA patients leads to a significant improvement in joint-related complaints, and thus, to an increase in patient quality of life. In contrast, the health-related behavior of the OA patients did not change. In particular, the sedentary behavior, which was the focus of attention of this study, did not show any postoperative change, even though short interruptions of the sedentary time were taken into account. The fact that long periods of uninterrupted sitting (≥30 min) remained unchanged after arthroplasty indicates a certain need for new interventions that have a lasting impact on patient SB. The relief of pain and the restoration of joint functionality may not be sufficient to achieve this.

## Figures and Tables

**Table 1:** Sociodemographic and clinical information of the OA patients.

Patient Data	Number or Mean (±SD)
Age [years]	68.88 ± 6.75
Gender	9 male, 7 female
BMI ^a^	26.39 ± 4.33
BMR [kcal] ^b^	1475.57 ± 320.20
Education ^c^	3 sl1, 10 sl2, 1 tl *
Professional status ^d^	13 r, 1 pt, 2 ft
Comorbidities ^e^	1.31 ± 0.79
Pain medication ^f^	1.94 ± 1.39
Lequesne index	9.81 ± 2.94
Follow-up (month postarthroplasty)	8.91 ± 2.30

^a^ Body mass index (body mass divided by the square of the body height [kg/m^2^]). ^b^ Daily basal metabolic rate (BMR) according to the gender specific Harris-Benedict energy equations. ^c^ Education levels: pl (primary level; age: 6–10 years), sl1 (secondary level I; age: 10–15/16 years), sl2 (secondary level; age: 15–19 years), tl (tertiary level; age > 19 years) ^d^ Professional status (r = retired; pt = part time job; ft = full time job) ^e^ Pathological conditions (cardiovascular, pulmonary, metabolic, gastrointestinal, liver, kidney, blood, cancer, depression, musculoskeletal diseases) (0 = best condition; 10 = worst condition) ^f^ Analgesic consumption related to osteoarthritis (0 = none | 1 = irregular | 2 = weekly | 3 = several times a week | 4 = daily). * data missing.

## Authors’ Note

The datasets generated and analyzed during the current study are not publicly available but are available from the corresponding author upon reasonable request.
